# Sleep and Temporal Lobe Epilepsy – Associations, Mechanisms and Treatment Implications

**DOI:** 10.3389/fnhum.2022.849899

**Published:** 2022-04-26

**Authors:** Divyani Garg, Laurel Charlesworth, Garima Shukla

**Affiliations:** ^1^Department of Neurology, Vardhman Mahavir Medical College and Safdarjung Hospital, New Delhi, India; ^2^Division of Sleep Medicine, Queen’s University, Kingston, ON, Canada; ^3^Division of Epilepsy and Sleep Medicine, Queen’s University, Kingston, ON, Canada

**Keywords:** refractory epilepsy, sleep, temporal lobe epilepsy, mechanisms, Epworth sleepiness scale, polysomnography, cognition

## Abstract

In this systematic review, we aim to describe the association between temporal lobe epilepsy (TLE) and sleep, with bidirectional links in mechanisms and therapeutic aspects. Sleep stages may variably impact seizure occurrence, secondary generalization and the development, frequency and distribution of interictal epileptiform discharges. Conversely, epilepsy affects sleep micro- and macroarchitecture. TLE, the most frequent form of drug resistant epilepsy (DRE), shares an enduring relationship with sleep, with some intriguing potential mechanisms specific to anatomic localization, linking the two. Sleep characteristics of TLE may also inform localizing properties in persons with DRE, since seizures arising from the temporal lobe seem to be more common during wakefulness, compared to seizures of extratemporal origin. Polysomnographic studies indicate that persons with TLE may experience excessive daytime somnolence, disrupted sleep architecture, increased wake after sleep onset, frequent shifts in sleep stages, lower sleep efficiency, decreased rapid eye movement (REM) sleep, and possibly, increased incidence of sleep apnea. Limited literature suggests that effective epilepsy surgery may remedy many of these objective and subjective sleep-related concerns, *via* multipronged effects, apart from reduced seizure frequency. Additionally, sleep abnormalities also seem to influence memory, language and cognitive-executive function in both medically controlled and refractory TLE. Another aspect of the relationship pertains to anti-seizure medications (ASMs), which may contribute significantly to sleep characteristics and abnormalities in persons with TLE. Literature focused on specific aspects of TLE and sleep is limited, and heterogeneous. Future investigations are essential to understand the pathogenetic mechanisms linking sleep abnormalities on epilepsy outcomes in the important sub-population of TLE.

## Introduction

Sleep is known to share a complex, profound, and bidirectional relationship with epilepsy. Non-rapid eye movement (NREM) and REM sleep exert variable influences on both temporal and extra-temporal epilepsy. Temporal lobe epilepsy (TLE), the most common form of drug-refractory epilepsy (DRE), shares a fascinating relationship with sleep.

Sleep facilitates generalization of focal seizures, particularly of temporal origin, as well as distribution based on localization of seizures (Bazil and Walczak, 1997; Crespel et al., 2000; Sinha et al., 2006). These, and additional sleep-related features, have been used to inform localization in pre-surgical assessment in drug-refractory epilepsy, in general (Singh et al., 2014; Narang et al., 2021). The reciprocal influence of epilepsy on sleep micro- and macroarchitecture has also been investigated, including TLE. However, a systematic review that included five studies after stringent selection, concluded that data was heterogeneous and limited (Sudbrack-Oliveira et al., 2019). Self-reported (subjective) and polysomnography (PSG) recorded (objective) sleep parameters seem to be more abnormal among persons with medically refractory epilepsy, including TLE (Zanzmera et al., 2012; Grigg-Damberger et al., 2020), which may improve with successful epilepsy surgery (Zanzmera et al., 2013). In persons with both refractory and medically controlled focal epilepsy, sleep abnormalities influence memory and executive function (Chakravarty et al., 2019).

It is important to highlight the growing corpus of data on TLE as a network disorder. This aids in our understanding of some of the commonalities that this specific epilepsy type shares with other focal onset epilepsy types, in terms of mechanisms linking it with sleep. González Otárula and Schuele (2020) have summarized neurophysiological, imaging and clinical data on brain networks in TLE. They demonstrate how recognizing the TLE associated network alterations can improve delineation of the epileptogenic zone (for epilepsy surgery), and also predict seizure outcome; while also providing multi-dimensional evidence on functional connectivity.

Despite pervasive interlinkage, a clear description of the multiple facets associating TLE and sleep are lacking. There is no comprehensive review elucidating potential underlying mechanisms, and clinical impact on patient treatment and prognosis of sleep issues in TLE. Hence, in this review, we aimed to explore the link between TLE and sleep, focusing on identifying mechanisms by which they exert reciprocal influence, and how these impact treatment paradigms.

## Methods

The Preferred Reporting Items for Systematic Reviews and Meta-Analyses (PRISMA) statement and guidelines were used to report this review.

We adopted a PubMed search strategy using the following terms: (”Temporal lobe epilepsy” AND “sleep”) OR (”Temporal lobe epilepsy” AND “sleep” AND “association”) OR (”Temporal lobe epilepsy” AND “sleep” AND “mechanism”) OR (”Temporal lobe epilepsy” AND “sleep” AND “treatment”) OR (”Temporal lobe seizure” AND “Sleep”) OR (“Temporal lobe epilepsy” AND “sleep” AND “Obstructive sleep apnea”) OR (“Temporal lobe seizure” AND “sleep” AND “Obstructive sleep apnea”) OR (“Temporal lobe epilepsy” AND “sleep” AND “SUDEP”) OR (“Temporal lobe epilepsy” AND “sleep” AND “SUDEP”). The search was conducted on 20th October 2021. We included studies in English language and those published after 1999. We selected studies based on (1) whether subject selection criteria clearly defined TLE, for clinical studies, and (2) if the study described aspects of sleep pertinent to TLE. We excluded descriptive reviews, and individual case reports. After excluding duplicates, titles and abstracts of studies were assessed by two reviewers (DG, LC). Any conflict was resolved in consultation with the third reviewer (GS). Full texts of studies were accessed and assessed for eligibility, and eligible studies were included in the final review.

### Data Extraction

We used a standardized data collection form to extract relevant information, where available. Data extraction was independently conducted by two reviewers (DG, LC) and cross-checked for errors. Any rectifications were made by consensus between the three reviewers (DG, LC, and GS). For all studies involving human subjects, variables extracted were: First author, year of publication, country, study design, study objective/s, inclusion criteria, exclusion criteria, study population, sleep instrument/s used, main findings, and effect size, if available. For all studies involving animal models, variables extracted were: First author, year of publication, country, study design, study objective/s, animal model used, intervention, sleep instrument, main findings, and effect size, if applicable.

### Assessment of Risk of Bias

Two reviewers (DG, LC) independently assessed quality appraisal of qualifying studies. A third reviewer (GS) evaluated the appraisal. Any conflict was resolved by consensus among the three reviewers. As the studies were heterogeneous in design, the risk of bias of individual studies was assessed using the National Heart, Lung and Brain Institute health quality appraisal tools, as per the Cochrane handbook recommendations (Higgins et al., 2011).

## Results and Discussion

**FIGURE 1:**
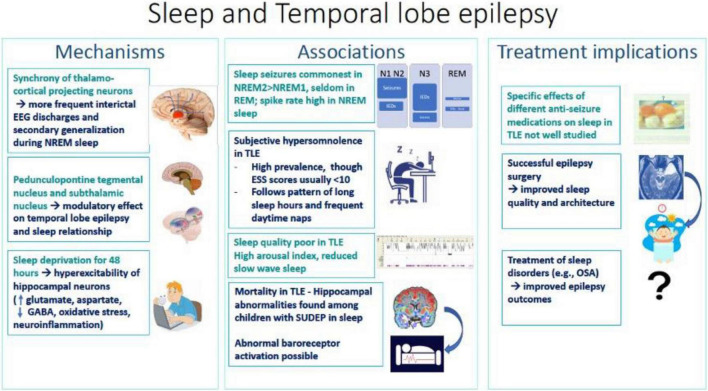
Summary diagram of associations, mechanisms and treatment implications of sleep and temporal lobe epilepsy.

This is a systematic review, among the first analyzing the association of TLE with sleep, focusing on mechanisms and modifier effects (Figure 1). Despite substantial literature on the effects of sleep and epilepsy on each other, the relationship between the most common form of DRE, TLE, and sleep, remains vastly underexplored.

**FIGURE 2:**
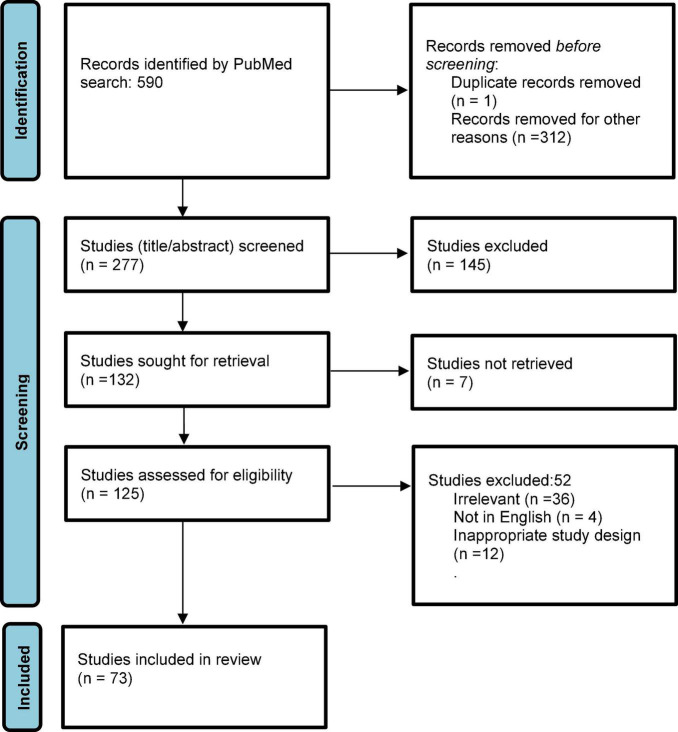
PRISMA Flow diagram of included studies.

The search yielded a total of 590 publications (Figure 2) on October 21, 2021. After applying filters of “English” and time range (beyond 1999) and removing duplicates, 277 studies entered screening. After screening title and abstracts, 145 studies were excluded, and 132 studies were accessed for full text. Of these, seven studies did not have full texts available, and were excluded. Of 125 full texts accessed, 52 studies were excluded due to wrong study design (descriptive reviews or single case report), not being in English, or irrelevant to the topic. Ultimately, 74 studies were included in the review. As per the quality assessment analysis, seven studies (9.5%) were judged to be “good,” 31 (41.8%) were “fair” and 36 (48.6%) were of “poor” quality. Most studies included in this review had small sample sizes and heterogeneous design.

### Mechanisms Interlinking Temporal Lobe Epilepsy and Sleep

Temporal lobe epilepsy is currently conceptualized as a network disease, due to modifications in functional connectivity in a widespread manner, between temporal and extratemporal brain regions. The mechanism underlying the increased occurrence of interictal epileptiform discharges (IEDs) during NREM sleep is neuronal synchrony of thalamo-cortical projecting neurons, that are necessary to maintain NREM sleep. This synchrony is the result of various neurochemical and neurophysiological processes that likely also facilitate IEDs. There is consensus that REM sleep, in which there is neuronal asynchrony, inhibits the propagation and maintenance of epileptiform discharges.

Shouse et al. (2000, 2004) studied the amygdala kindling feline model of TLE, to assess the role of neural generators in provocation of seizure discharges during NREM, and inhibition during REM sleep. Synchrony of EEG oscillations as determined by tonic slow waves and phasic arousal events, promoted seizure propagation during NREM sleep, in combination with anti-gravity muscle tone which facilitates motor seizure component. Similarly, loss of synchrony during awake state and REM sleep were observed to decrease electrographic seizure discharges, and atonia during REM sleep, to inhibit seizure-related movements.

At the ponto-mesencephalic junction, two groups of cholinergic neurons exist, in the lateral dorsal tegmentum (LDT) and pedunculopontine tegmentum. These activate neurons (“effector cells”) in the pontine reticular formation and mesencephalic reticular formation (MRF), responsible for REM features, including rapid eye movement, muscle atonia, and low-voltage fast EEG activity (Ng and Pavlova, 2013). MRF neurons largely depolarize the thalamus, leading to transmission of information to the cortex, and resultant desynchronization. In contrast, cholinergic neurons are less active in NREM sleep, particularly slow wave sleep. The inactive thalamus thereby permits innate synchrony of cortical firing. Hence, the asynchrony of REM state prevents spatial and temporal summation of spontaneous depolarization of neurons, believed to underlie focal epilepsy and IEDs (Badawy et al., 2009). REM sleep also inhibits spread *via* long association pathways, that contribute to seizure generalization (Ng and Pavlova, 2013). The pedunculopontine tegmental nucleus (PPTg) has been implicated in a REM sleep-promoting role. The mesopontine tegmentum modulates REM sleep *via* interactions between REM-inducing and REM-inhibiting neurons (Lim et al., 2009), and its role has been explored in DRE (Xu et al., 2015). A connection crucial for modulation of REM sleep seems to exist between the PPTg, particularly cholinergic neurons, and the subthalamic nucleus (STN), and involves melano-cortinergic signaling. Central melano-cortinergic signaling plays a key role in sympathetically mediated glucose homeostasis *via* STN-PPTg circuitry, as does sleep. Data from animal models supports the role of the PPTg and STN in modulating the relationship between sleep and epilepsy (Matos et al., 2010a). The STN, which is an output pathway of the basal ganglia, is known to exert modulatory influence on drug-resistant seizures. Chronic high-frequency stimulation of the STN led to a reduction in seizure frequency of penicillin-induced focal motor seizures, by disruption cortical synchronization in a primate model (Prabhu et al., 2015). Bilateral allo-transplantation of GABAergic cell lines into the substantia nigra pars reticulata in a rat model of TLE was demonstrated to be anti-epileptogenic (Nolte et al., 2008). However, clinical studies have reported increased spiking rates in certain subgroups of TLE patients during REM sleep and wake state (Malow et al., 1998; Scarlatelli-Lima et al., 2016). Although the neural basis of this remains unclear, the occurrence of focal IEDs during the REM state have been reported to demonstrate high concordance with localization or neuroimaging findings in some studies (Sammaritano et al., 1991; Malow et al., 1999; Singh et al., 2014; Scarlatelli-Lima et al., 2016; Yuan and Sun, 2020). A study by Singh et al. (2014), however, found that REM IEDs were no better defined and fewer in number, compared to those recorded in NREM sleep. Hence, whether IEDs in REM sleep have higher localizing value than those during NREM sleep, remains uncertain.

In a pilocarpine-induced rat model of TLE, sleep fragmentation was discernible (Matos et al., 2010b). The relaxed awake state, which marks the transition from wakefulness to sleep and the onset of synchronization mechanisms, was increased. The resting awake state specifically increases sensitivity to specific seizure types that depend on synchronization mechanisms (Shouse et al., 2000).

Hippocampal-cortical interactions are crucial for memory. Beenhakker and Huguenard elegantly posited that epileptic circuits “hijack” normal brain networks to produce seizures, such as the replacement of thalamo-cortical spindle generation circuits by spike and wave discharges (Beenhakker and Huguenard, 2009). Other authors have also reported data from animal models of TLE, showing that IEDs are coordinated with prefrontal spindle oscillations, bypassing the normal ripple-delta-spindle coordination that promotes physiological memory consolidation (Gelinas et al., 2016; Mendes et al., 2021).

Among persons with epilepsy (PWE), the presence of structural hippocampal abnormalities, in the form of hippocampal atrophy and mesial temporal sclerosis, probably modulate sleep and memory. The association between hippocampal lesions and memory impairment has been reported in several studies, leading to impaired storage of new memories (Mormino et al., 2009; Planche et al., 2017; Duff et al., 2020).

Not only are IEDs and seizures inter-linked with the sleep–wake cycle, but there is also evidence of overlying circadian rhythmicity. This seems to be more impactful on seizure occurrence, whereas IEDs are more influenced by sleep–wake state. It has been well-recognized that seizures follow a circadian rhythmicity, an observation that has gathered more robust data with the wider use of long-term intracranial recording devices. This is referred to as “seizure chronotype,” and is present in as much as 90% of PWE (Karoly et al., 2021).

In summary, the mechanisms interlinking TLE with different sleep stages may not be unique, at least as far as focal onset epilepsy is concerned, and largely involve the interplay between cholinergic inputs from the PPTg and LDT to the thalamo-cortical circuits, modulating innate cortical synchrony. The relationship of the mesial temporal region with the brainstem and thalamic centers possibly underlies memory deficits linked with TLE, as a result of “bypassing” of the thalamo-cortical spindle generation circuits that contribute to memory consolidation.

### Association Between Temporal Lobe Epilepsy and Sleep

#### Effects of Sleep on Temporal Lobe Epilepsy

##### Distribution of Seizures and Interictal Epileptiform Discharges

Sleep is known to facilitate the appearance of IEDs, particularly slow wave sleep (SWS) in patients with TLE (Sammaritano et al., 1991; Malow et al., 1999; Foldvary-Schaefer and Grigg-Damberger, 2006). Minecan et al. (2002) reported that epileptic seizures resembled IEDs in being more frequent during NREM than REM sleep, in a mixed population of patients with DRE undergoing pre-surgical evaluation. Specifically in TLE, we identified seven studies that supported the role of sleep in enhancing EEG-based localization of seizures and/or IEDs (Table 1; Herman et al., 2001; Clemens et al., 2003, 2005; Buechler et al., 2008; Singh et al., 2014; Yuan and Sun, 2020; Narang et al., 2021).

**TABLE 1:** Studies assessing localizing properties of sleep in TLE.

Author, year	Study design	Number of patients/seizures	Instruments used	Salient observations
Herman et al., 2001	Prospective observational	133 DRE patients with focal epilepsy, 86 with TLE	Video EEG with periorbital and chin EMG, and scalp/sphenoidal electrodes A subset of 34 patients with TLE underwent PSG	Temporal lobe seizures, especially neocortical temporal lobe seizures, were more common during wakefulness; seizures most likely to begin in NREM2.
Clemens et al., 2003	Cross-sectional	12 DRE MTLE	Video EEG with combined scalp and FoE electrodes	Scalp spiking showed an increase during SWS (NREM3, NREM4). FoE spiking showed an increase during NREM2.
Clemens et al., 2005	Cross-sectional	38 DRE TLE	Video EEG	Highest spike rates observed during NREM 3 and 4, followed by NREM2 and wake with eyes open. Spiking rate during REM was higher if the patient had secondarily generalized seizures.
Buechler et al., 2008	Retrospective	28 TLE patients seizure free post-surgery, 134 seizures	Video EEG	Ictal EEG during sleep was 2.5 times more likely to show focality of onset, compared to ictal EEG during wake state, and four times more likely to localize seizure onset (*p* = 0.04)
Singh et al., 2014	Prospective observational	70 patients with DRE; 64.2% were TLE	Video EEG with polygraphy	In patients who had both sleep and wake seizures recorded, sleep ictal and interictal EEG had higher localizing value
Yuan and Sun, 2020	Retrospective	59 patients with DRE; 37 with TLE		REM IEDs had higher localizing property than IEDs in wakefulness or NREM sleep. Accuracy for TLE was 90%, compared to 45% for ETLE
Narang et al., 2021	Retrospective	175 DRE	Video EEG	Temporal lobe seizures more frequent during wakefulness (77%) compared to extra-temporal (65%), *p* < 0.0001. Lateral temporal lobe seizures (88.6%) occurred more frequently during wakefulness compared to mesial temporal (75.5%), *p* = 0.0003

*DRE, drug refractory epilepsy; EEG, electroencephalography; EMG, electromyogram; ETLE, extra temporal lobe epilepsy; FoE, Foramen ovale electrodes; MTLE, mesial temporal lobe epilepsy; PSG, polysomnography; TLE, temporal lobe epilepsy.*

Herman et al. (2001) conducted a prospective observational study among 133 patients with medically refractory focal epilepsy who were candidates for epilepsy surgery. Sleep seizures were found to be more frequent during NREM2 (68%) and NREM1 (23%) whereas none occurred during REM sleep. In another study by Clemens et al. (2003) using combined scalp and foramen ovale electrodes (FoE), spiking rates were significantly increased in NREM3 and 4 (SWS) using scalp electrodes, and during NREM2 using FoEs. This surface-deep discrepancy was explained by the authors with the hypothesis that spikes originating in the mesio-temporal region propagated to the lateral temporal cortex and appeared as FoE dependent scalp spikes. In another video EEG-based study among 38 TLE patients, most (65.8%) patients demonstrated highest spiking in NREM3 and 4 (SWS) (Clemens et al., 2005). Spiking rates correlated with duration of epilepsy. Another interesting finding in this study was that relative spike density during wake state with eyes closed was higher among patients with hippocampal sclerosis compared to those with normal MRI.

Buechler et al. (2008) compared 58 sleep seizures with 76 awake seizures among 28 TLE patients who had been seizure free for at least 2 years after anterior temporal lobectomy. Sleep seizures were found to be more potently localizing. The odds of accurate localization in sleep seizures were 4.15 (1.05–16.33) (*p* = 0.04). Sleep seizures were also 2.58 times more likely to be focal. Similar observations were made by Singh et al. I2014). Among patients with DRE who had both sleep and wake seizures recorded, sleep seizures were twice as localizing as wake seizures. Narang et al. (2021) using video EEG among patients with drug refractory focal epilepsy, observed the propensity for temporal lobe seizures to occur more frequently during wakefulness, as compared to seizures originating from extra-temporal brain regions.

Interestingly, Yuan and Sun (2020) observed the higher localizing value of IEDs during REM in TLE, compared to NREM or wakefulness.

Overall, seizures and IEDs in TLE have been reported to occur more frequently in NREM sleep, compared to REM sleep. Sleep seizures also been observed to exhibit higher localizing properties. Although seizures in TLE tend to occur rarely in REM sleep, in most studies, these displayed enhanced localizing properties in one study in our review. Definite inter-individual variability in spiking rates and the use of anti-seizure medications (ASMs) may partially explain these discrepant findings. Moreover, longer duration of epilepsy and the presence of hippocampal sclerosis were factors correlating with increased localization, but further associations, including sleep and epilepsy-related properties, must be delineated in future studies, to increase the yield of electrophysiological investigations.

##### Generalization of Temporal Lobe Onset Seizures in Sleep

Four studies have assessed the secondary generalization of seizures in sleep (Table 2; Herman et al., 2001; Jobst et al., 2001; Sinha et al., 2006; Narang et al., 2021). Herman et al. (2001) prospectively studied 133 patients *via* video EEG, with 34 undergoing overnight PSG. Secondary generalization of temporal lobe seizures was more frequent during sleep (31%) than wakefulness (15%) (Herman et al., 2001). Jobst et al. (2001) conducted a cross-sectional study among 29 patients with MTLE who were undergoing pre-surgical evaluation. Of the 286 seizures, 69 had secondary generalization; and among these, 60.9% (*n* = 42) occurred out of sleep. Sinha et al. (2006) assessed seizures among 57 patients with DRE (42 TLE), who had seizures both in sleep and wakefulness. Secondary generalization was significantly more frequent during sleep than wakefulness, although differences in semiology were not observed (Sinha et al., 2006). Secondary generalization relies on thalamo-cortical interactions, which are facilitated by NREM sleep. While all four studies reported secondary generalization to occur more frequently during sleep, predilection for specific sleep stages was not elucidated. No instances of generalization were reported during REM sleep in any of these studies.

**TABLE 2:** Studies assessing secondary generalization of TLE in sleep.

Author/Year	Study design	Number of patients	Sleep instrument	Outcomes
Herman et al., 2001	Prospective observational	133 DRE patients, 86 with TLE	Video-EEG with periorbital and chin EMG, and scalp/sphenoidal electrodes A subset of 34 patients with TLE underwent PSG	Temporal lobe seizures more likely to generalize during sleep (31%) than wakefulness (15%)
Jobst et al., 2001	Cross-sectional	29 MTLE	Video-EEG	Secondary generalization more likely to occur out of sleep (60.9%) than wakefulness (43.3%) (*p* < 0.01)
Sinha et al., 2006	Retrospective	57 DRE of which 42 were TLE	Video-EEG	Secondary generalization more likely to occur in sleep than wakefulness (*p* < 0.01)
Narang et al., 2021	Retrospective	175 DRE	Video-EEG	Secondary generalization during sleep higher in temporal lobe onset seizures, compared to extra-temporal lobe onset seizures

*DRE, drug refractory epilepsy; EEG, electroencephalography; MTLE, mesial temporal lobe epilepsy; TLE, temporal lobe epilepsy.*

##### Effects of Sleep Deprivation on Seizures and Electroencephalography in Temporal Lobe Epilepsy

Sleep deprivation has been recognized as a maneuver to enhance diagnostic yield of IEDs in focal epilepsy, to the extent of 40% (Giorgi et al., 2013). However, studies in TLE are limited, and most have studied mixed populations. The mechanism underlying this activating phenomenon is believed to go beyond mere sleep induction following sleep deprivation. Microstructural sleep alterations may play a role, as indicated in a retrospective study by Giorgi et al. (2017). In this study, sleep microarchitecture was compared between nocturnal physiological sleep and morning sleep following sleep deprivation in 13 patients with TLE. Increased CAP rate, and increased sleep instability, was demonstrated on the sleep deprived EEG, along with increased spike index during NREM sleep, which may partly explain the activating influence of sleep deprivation on IED yield. IEDs were strongly associated with A1 and A2 phases during the first NREM/REM cycle, in this study. In a pilocarpine rat model of TLE, paradoxical sleep deprivation for 48 h induced a state of hyperexcitability in the hippocampus, indicated by significantly increased levels of excitatory amino acids (glutamate, aspartate), and decrement in inhibitory amino acid, GABA, and oxidative stress and neuroinflammation (Aboul Ezz et al., 2021). These may indicate increased susceptibility to seizures following sleep deprivation. An fMRI-based study showed altered functional activity in the region of epileptiform discharges after sleep deprivation in patients with TLE (Cartella et al., 2019).

#### Effects of Temporal Lobe Epilepsy on Sleep

##### Subjective Hypersomnolence

In general, excessive sleepiness is highly prevalent among persons with epilepsy with nearly 78% reporting feeling excessively sleepy during the day (Khatami et al., 2006; Chen et al., 2011; Grigg-Damberger et al., 2020); with 24% scoring a 10 or higher score on the Epworth Sleepiness scale (ESS) in a mixed cohort (Grigg-Damberger et al., 2020).

Five studies have assessed subjective hypersomnolence among persons with TLE (Table 3) using ESS (Carrion et al., 2010; Zanzmera et al., 2012; Nayak et al., 2016; Scarlatelli-Lima et al., 2016; Yaranagula et al., 2021). Of these, the study by Zanzmera et al. (2012, 2013) enrolled a cohort of DRE patients, the majority being patients with TLE. Subjective EDS was reported by 45% of patients and mean ESS scores were 6.9 ± 3.5.

**TABLE 3:** Studies assessing subjective hypersomnolence among persons with TLE.

Author, year of publication	Study design	Number/profile of patients	Metrics used for subjective hypersomnolence	Outcome
Yaranagula et al., 2021	Prospective observational	40 patients with DRE TLE-HS of whom 20 underwent epilepsy surgery	ESS PSQI	3.45 ± 1.8 (range: 0–6); post-op: 3.05 ± 2.5 (range: 0–8); *P* = 0.48 3.63 ± 1.3 (1–7) versus 2.48 ± 1.5 (0–7) #
Nayak et al., 2016	Cross-sectional	20 patients with Drug-naïve TLE, 20 TLE on carbamazepine, and 40 healthy controls	ESS PSQI NCSDQ	Mean ESS scores 5.50 ± 3.95 Mean score = 3.95 ± 2.68# Higher prevalence of complaints compared to sleep maintenance insomnia (*p* = 0.029) and nocturnal awakening to pass urine (*p* = 0.048) #
Scarlatelli-Lima et al., 2016 (Scarlatelli-Lima et al., 2016)	Cross-sectional	38 patients with DRE TLE-HS	ESS PSQI SSS	Mean score 7.7 ± 4.5 (9; 16.1% reported ESS > 10) 5.3 ± 2.8; (15; 27% reported PSQI > 5) 1.5 ± 0.9 (4; 7.1% reported SSS < 2)
Zanzmera et al., 2012, 2013	Prospective	DRE (most were TLE) – 20 patients, and 20 patients with medically controlled epilepsy	ESS	Mean score 6.9 ± 3.5; ESS > 10 in 6 (30%); EDS reported by 9 (45%) patients Self-reported sleep parameters worse in DRE versus controlled epilepsy group
Carrion et al., 2010	Prospective observational	48 patients with DRE TLE who underwent epilepsy surgery, compared to 43 patients with non-surgical refractory TLE	ESS PSQI	ESS scores varied from 0 to 20 before surgery and from 0 to 14 After surgery, representing a significant reduction after surgery (*P* < 0.001). 5.65 (3.71) at baseline, 2.25 (3.50) at 3 months, 1.48 (2.02) at 1 year; *p* < 0.001

*#Statistically significant compared to controls.*

*DRE, drug refractory epilepsy; ESS, Epworth Sleepiness Scale; HS, hippocampal sclerosis; NCSDQ, NIMHANS Comprehensive Sleep Disorders Questionnaire; PSQI, Pittsburgh Sleep Quality Index; SSS, Stanford sleepiness scale; TLE, temporal lobe epilepsy.*

Mean ESS scores in all the five studies were reported to be below 10. Clinically significant excessive daytime sleepiness, defined as ESS > 10, was reported by 16.1% of patients in the study by Scarlatelli-Lima et al. (2016). However, this study lacked a control population. Nayak et al. (2016) and Yaranagula et al. (2021) reported comparable ESS scores between patients and controls. Both Carrion et al. (2010) and Zanzmera et al. (2012) used epilepsy patients as controls. However, whereas Zanzmera et al. (2012) employed medically controlled epilepsy patients, and showed significant difference with refractory TLE in sleep complaints, Carrion et al. (2010) used non-surgical refractory TLE patients, who did not differ from surgical candidates in subjective sleep parameters.

An important point about daytime hypersomnolence in epilepsy emerges from the data reported by Zanzmera et al. (2012) Despite mean ESS scores not being high, 75% patients in the DRE group and 40% in the controlled epilepsy group reported daytime naps and slept for a median of 10.5 (range 8–15) and 9 (7–11) hours per 24-h cycle, respectively. The number and category of ASMs that most patients included in most of these studies also likely indicates a relationship with subjective daytime sleepiness, as well as total time spent sleeping over an average 24-h cycle. Therefore, the pattern of daytime hypersomnolence in epilepsy is different from that observed in primary sleep disorders and appears to manifest more in terms of long sleep hours and/or napping during the day.

##### Self-Reported Sleep Quality

Patients with TLE report poorer sleep quality. In a study by Yaranagula et al. (2021), 40 patients with drug refractory TLE with hippocampal sclerosis demonstrated poorer scores on Pittsburgh Sleep Quality Index (PSQI) [3.63 ± 1.3 (1–7) versus 2.48 ± 1.5 (0–7); *p* = 0.0004], and NIMHANS Comprehensive Sleep Disorders Questionnaire, compared to controls. PSQI assesses overall sleep quality, and a score > 5 on PSQI is considered significant. Only three patients and two controls had PSQI > 5 in this study. In the study by Scarlatelli-Lima et al. (2016), 27% reported PSQI > 5.

In the study by Zanzmera et al. (2012), multiple self-reported sleep parameters (including total sleep time in 24 h, sleep duration in daytime, sleep duration at night, daytime nap, EDS, ESS, and average sleep time in the previous week) were significantly worse in patients with DRE compared to medically controlled epilepsy group.

Although patients with TLE have been reported to suffer excessive daytime somnolence, two controlled studies did not demonstrate a significant difference from healthy controls in ESS scores. Mean ESS scores in studies included also did not enter pathological range. The reason for lower overall scores, particularly in the study by Yaranagula et al. (2021) may be dependent partly on the seizure frequency. The seizure frequency was higher in the studies by Carrion et al. (2010) (7.83 ± 0.97), Zanzmera et al. (2012) (60/month), Nayak et al. (2016) (10.5 ± 22.67/month), and Scarlatelli-Lima et al. (2016) (11.9 ± 20.6/month) compared to Yaranagula et al. (2021) (3.5 ± 4.7/month).

Subjective sleep quality on PSQI was significantly impaired among patients with TLE compared to healthy controls in two of the studies (Nayak et al., 2016; Yaranagula et al., 2021). Another interesting observation in two studies was that although patients with DRE-TLE self-reported longer TST, actual recorded TST was shorter, indicating that TLE patients *believe* that they spend more time sleeping (Zanzmera et al., 2012; Chakravarty et al., 2019). This could also reflect the poor sleep quality resulting in increased amount of time spent in bed, higher percentage of patients napping during the daytime and overall more time spent sleeping during the day. The insignificant differences in ESS scores found in different studies could be attributed to the pattern of excessive daytime sleepiness in this population, which is more in the form of long sleep hours, and higher number and duration of daytime naps, especially naps immediately following intake of medications.

##### Temporal Lobe Epilepsy and Sleep Macro-Architecture

Sleep macrostructure or macro-architecture refers to global organization of sleep and does not deal with brief arousals that occur during sleep. Nine studies have assessed the impact of TLE on sleep macro-architecture (Table 4; Crespel et al., 2000; Serafini et al., 2012; Zanzmera et al., 2012, 2013; Nayak et al., 2016; Scarlatelli-Lima et al., 2016; Giorgi et al., 2017; Chakravarty et al., 2019; Yaranagula et al., 2021).

**TABLE 4:** Studies assessing sleep macro-architecture in TLE.

Sleep parameter	Crespel et al., 2000	Serafini et al., 2012 (*N* = 11)	Zanzmera et al., 2012 (*N* = 20)	Zanzmera et al., 2013 (*N* = 17)&	Nayak et al., 2016 (*N* = 20)$	Scarlatelli-Lima et al., 2016 (*N* = 16)	Giorgi et al., 2017 (*N* = 13)	Chakravarty et al., 2019 (*N* = 37 with DRE)	Yaranagula et al., 2021 * (*N* = 22)
Study design	Prospective observational- Two studies	Prospective observational pre- post	Prospective observational	Prospective observational	Cross-sectional	Cross-sectional	Retrospective	Prospective observational	Prospective observational
Study population	First study: 15 DRE TLE Second study: 90 patients with pure MTLE and 20 with mesiolateral and lateral TLE	Refractory MTLE	DRE (most were TLE) – 20 patients, and 20 patients with medically controlled epilepsy	DRE (most were TLE)	Three groups: Drug-naïve TLE (20), TLE on CBZ (20), and healthy controls (40)	Refractory mesial TLE-HS	TLE	37 with refractory focal epilepsy, 37 with medically controlled focal epilepsy, and 40 age and sex-matched controls	Refractory TLE with unilateral HS
TIB (min)	–	–		–	487.2 ± 39.6	–	–	–	502.81 ± 50.16 (360–601.4)#
TST (min)	–	355.34 ± 79.68	340.4 (147–673)	322.2 ± 74.4	378.6 ± 91.2	390 ± 54	88.3 ± 20.8	371.97 ± 94.22	421.40 ± 93.36 (97–527.5)#
Sleep efficiency (%)	–	79.11 ± 17.87	80.5 (40.5–98.0)	77.05 ± 14.25	77.39 ± 15.86#	86.1 ± 9.8	–	76.25 ± 17.12	82.86 ± 15.05 (26.9–99.1)
Wake (%)	–	–		–	24.69 ± 19.45#	–	–	–	–
N1%	–	12.04 ± 9.77		11.29 ± 7.59	6.38 ± 3.54#	7.5 ± 4.6	8.2 ± 5.4	22.91 ± 16.51	21.72 ± 11.02 (7.2-2.2) #
N2%	–	47.26 ± 12.09		49.57 ± 11.10	40.66 ± 13.63	49.0 ± 10.6	44.2 ± 16.6	52.86 ± 16.59	52.98 ± 16.67 (28.8-88.5) #
N3%	–	23.15 ± 9.17		18.29 ± 8.83	19.77 ± 8.56	26.6 ± 11.8	36.3 ± 14.5	14.37 ± 15.11	12.6 ± 8.89 (0.1–30) #
REM%	–	17.53 ± 5.78		22.29 ± 3.77	15.45 ± 9.96	16.7 ± 6.6	11.3 ± 9.2	8.37 ± 7.7	12.67 ± 7.05 (0–26.3)
NREM%	–	–		–	66.89 ± 9.93	–	–	–	–
WASO	–	87.47 ± 75.35	19.5 (1–56)	24.42 ± 14.82	–	17.4 ± 15.6	26.0 ± 33.6	18.21 ± 12.88	–
Sleep onset latency (min)	–	29.21 ± 28.91	14 (4.0–112.5)	13.67 ± 10.82	19.57 ± 21.36	28.1 ± 25.3	–	19.79 ± 25.95	10.47 ± 10.56 (0.5–45.5)
Mean arousal index	–	–	10.0 (0–31.4)	12.41 ± 8.91	11.56 ± 6.51	11.5 ± 6.6	-	13.26 ± 9.87	7.2 ± 3.13 (3.2e14)
REM arousal index	–	–		–	12.17 ± 10.04	–	–	–	–
NREM arousal index	–	–		–	11.00 ± 6.87	–	–	–	–
PLM index	–	–	0.3 (0–2.4)	0.60 ± 0.82	5.19 ± 8.47	–	–	–	0.28 ± 0.87 (0–3.9) #
AHI	–	–	1.22 (0–11.93)	2.95 ± 3.53	0.46 ± 0.67	–	–	3.65 ± 6.46	2.91 ± 3.17 (0.1–15.2) #

*Number of patients whose overnight PSG amenable for analysis.

*#Statistically significant compared to healthy controls.*

*$Drug-naïve TLE.*

*&Pre-surgical measurements.*

*AHI, apnea hypopnea index; CBZ, carbamazepine; DRE, drug refractory epilepsy; HS, hippocampal sclerosis; NREM, non-rapid eye movement; PLM, periodic limb movement; REM, rapid eye movement; TIB, time in bed; TLE, temporal lobe epilepsy; TST, total sleep time; WASO, wake after sleep onset.*

In the study by Yaranagula et al. (2021) analysis of sleep architecture on polysomnography (PSG) revealed that DRE patients with TLE with hippocampal sclerosis (HS) slept longer but had lower sleep efficiency compared to healthy controls. No significant change in sleep parameters at 3 months was noted among 20 patients who underwent epilepsy surgery in this study. However, the mean duration of post-operative follow up was only 5.25 ± 2.4 months. PSG analysis showed that sleep architecture was disturbed, with increase in awakenings and arousals, longer N1 and N2 and shorter slow wave sleep (SWS). Apnea indices, including central, mixed, hypopnea and apnea-hypopnea, were higher in patients. Patients on ≥ 3 ASMs demonstrated lower sleep efficiency. Chakravarty et al. (2019) demonstrated that total sleep time, sleep efficiency, sleep latency and REM latency were significantly worse among patients with DRE compared to medically controlled epilepsy. In another study by Scarlatelli-Lima et al. (2016) among refractory mesial TLE patients with HS, disruption of sleep architecture was observed, with increased sleep fragmentation, increased WASO and N1 and N3 sleep, reduced REM sleep and abnormal REM latency. Nayak et al. (2016) demonstrated decrease in sleep efficiency, and increased wake and N1 among patients with TLE compared to controls, although this study primarily aimed to assess sleep microstructure in drug-naïve TLE versus TLE on carbamazepine (CBZ) treatment versus controls. Giorgi et al. (2017) also studied sleep architectural differences between nocturnal PSG compared to sleep deprived EEG in patients with TLE, and found increase in sleep instability in the latter. In the study by Zanzmera et al. (2012, 2013) patients with DRE demonstrated lower total sleep time and sleep efficiency, and increased arousals and WASO compared to medically controlled epilepsy. Similarly, in an older study, Crespel et al. (2000) demonstrated disruption in sleep architecture and decreased sleep efficiency among patients with TLE.

Laterality of TLE may also impact sleep microarchitecture. In a retrospective study by Nakamura et al. (2017) among 16 patients with TLE (10 had left TLE and six had right TLE), the percentage of REM sleep was observed to be significantly lower (median 8.8%, interquartile range 5.5–13.8%) among patients with left compared to right TLE (median 17.0%, 14.1–18.3%).

##### Temporal Lobe Epilepsy and Sleep Microarchitecture

Sleep microarchitecture or microstructure refers to brief transient events that occur during sleep, including arousals, and cyclical alternating pattern (CAP). Seizures and IEDs are more common in phase A and non-CAP phase in generalized and focal epilepsy, although TLE patients have not been specifically analyzed in these studies (Parrino et al., 2000; Manni et al., 2005; Benbir et al., 2013). Three studies were found to assess sleep microstructure in TLE (Table 5).

**TABLE 5:** Studies assessing sleep microarchitecture in TLE.

	Nayak et al., 2016 *N* = 20$	Giorgi et al., 2017 *N* = 13	Romigi et al., 2020 *N* = 10
Study design	Cross-sectional	Retrospective	Prospective observational
Study population	Three groups: Drug-naïve TLE (20), TLE on CBZ (20), and healthy controls (40)	TLE	TLE
CAP rate%	54.94 ± 3.61	61.4 ± 10.9	41.67 ±31.26
CAP rate N1%	64.94 ± 18.50	25.3 ± 27.3	41.76 ±15.53#
CAP rate N2%	56.46 ± 8.18	51.0 ± 12.4	33.01 ±13.72
CAP rate N3%	47.04 ± 14.36	84.4 ± 14.3	61.55 ±21.26
Phase A1 index (n/h)	26.42 ± 5.91	59.1 ± 20.5	33.23 ±11.33
Phase A2 index (n/h)	26.42 ± 5.91	18.1 ± 4.9	13.01 ±4.84
Phase A3 index (n/h)	16.13 ± 7.54	6.5 ± 5.1	6.25 ±2.89#
CAP cycle index (n/h)	62.65 ± 8.66	–	–
CAP cycle duration (s)	32.01 ± 3.75	29.7 ± 4.3	33.54 ±1.61
Phase A duration (s)	19.42 ± 3.53	–	8.14 ±0.57#
Phase B duration (s)	12.58 ± 3.08	18.5 ± 4.5	25.53 ±1.57
Phase A1 duration (s)	12.58 ± 3.08	9.8 ± 0.9	7.2 ±0.80
Phase A1%	42.69 ± 9.83	–	58.74 ±7.19#
Phase A2 duration (s)	18.60 ± 6.83	15.4 ± 3.0	8.21 ±0.77
Phase A2%	32.00 ± 8.38	–	25.56 ±5.25
Phase A3 duration (s)	21.53 ± 4.30	15.1 ± 5.9	11.59 ±1.36#
Phase A3%	25.30 ± 9.76	–	15.7 ±5.89#
CAP sequence index	31.32 ± 4.33	–	–
CAP sequences (n)	–	8.5 ± 3.6	37.9 ±7.57
Sequence duration (s)	–	397.5 ± 188.6	227.42 ±49.86

*$Drug naïve TLE.*

*#Statistically significant compared to controls.*

*CAP, cyclic alternating pattern; CBZ, carbamazepine; DRE, drug refractory epilepsy; TLE, temporal lobe epilepsy.*

Giorgi et al. (2017) demonstrated strong association between IEDs and CAP A1 and A2 phases in morning sleep after sleep deprivation in patients with TLE. Nayak et al. (2016) showed increase in REM arousal indices and overall CAP rates among TLE patients. Romigi et al. (2020) observed significantly higher N1 CAP rate, lower phase A1 (%), higher phase A3 (%), higher duration of phase A and A3, and higher A3 index compared to controls. This study was mainly aimed at observing effects of eslicarbazepine on sleep profile in TLE. Although eslicarbazepine did not induce significant changes in the PSQI score or sleep macrostructure, sleep microstructure showed improvement.

The above findings are interesting because phase A is the major gateway, both for IEDs and seizures (Parrino et al., 2012). A3 is also associated with cognitive impairment. Another study observed an association between cognitive phenotypes in TLE and sleep spindle- K complex density; density of spindle-K complex was found to be lowest among TLE patients with memory and language impairment, and low among patients with memory impairment compared to those with language impairment alone (Gupta et al., 2019).

In summary, studies based on PSG analysis of sleep macrostructure definitively demonstrated reduced sleep efficiency and disrupted sleep architecture among patients with TLE. N1 and/or N2 duration is increased, along with findings of increased WASO and slow wave sleep and reduced REM sleep duration. These findings are in line with what has already been observed among patients with DRE, in general (Pereira et al., 2012; Carotenuto et al., 2014). It is clear that poor seizure control in DRE is associated with altered sleep architecture and reduced sleep efficiency, due to multiple factors like seizure related arousals, co-morbid psychiatric and sleep disorders, common etiological factors and medication effects. However, sleep structure has been found to be much worse (in the form of significantly higher WASO values) particularly among patients with DRE-TLE, as compared to DRE-frontal lobe epilepsy (FLE) and healthy controls, as reported in a recent systematic review analyzing sleep architecture among patients with DRE (Sudbrack-Oliveira et al., 2019). One of the factors responsible for this specific difference discussed, is the comparatively lower seizure frequency in the DRE-TLE group. However, the difference is more likely due to varying pathophysiology and disease mechanisms in TLE vs. FLE.

##### Sleep Apnea in Temporal Lobe Epilepsy

An intriguing relationship seems to exist between TLE and sleep apnea. In a questionnaire-based study by Yildiz et al. (2015), prevalence of sleep disorders was compared between patients with TLE (*n* = 101) and ETLE (*n* = 88) admitted to an epilepsy monitoring unit. The instruments used to assess the prevalence of sleep disorders included medical outcome study-sleep scale (MOSS), Epworth sleepiness scale (ESS), and sleep apnea scale of the sleep disorders questionnaire (SD-SDQ). SD-SDQ scores were significantly higher among patients with TLE, suggesting an increased reported risk of obstructive sleep apnea among these patients. No objective confirmation through PSG was available from this study. Sivathamboo et al. (2019) studied 255 patients diagnosed to have epilepsy (among 370 patients admitted for inpatient video-EEG monitoring) and found 67 among these (26.3%) to have moderate to severe sleep disordered breathing (SDB) (apnea hypopnea index > 15). Independent predictors for this were older age and body mass index (BMI). In an older pilot study, Malow et al. (2008) assessed the effect of treating OSA with continuous positive airway pressure (CPAP) on adult patients with DRE. Among 45 patients who met inclusion criteria and underwent PSG, 36 (80%) met PSG criteria for OSA, with a baseline mean AHI ranging between 16 and 19 in the two groups (receiving treatment with CPAP and sham CPAP respectively). In the study by Zanzmera et al. (2012), among patients with medically refractory epilepsy, most of whom were patients with TLE, 4/20 (20%) patients were reported to have an apnea-hypopnea index (AHI) > 5, compared to none in the medically controlled group (*p* < 0.01). A significantly higher number of desaturations was also observed in the refractory group.

Obstructive sleep apnea may be associated with sudden unexpected death in epilepsy patients (SUDEP) risk in patients with refractory epilepsy (McCarter et al., 2018), although conflicting data exists (Cheng, 2021; Phabphal et al., 2021). Lack of heart rate variability during sleep apneas in patients with TLE has been proposed as an indirect marker of SUDEP (Nayak et al., 2017).

Diminished hippocampal and mamillary body volume among patients with obstructive sleep apnea has been observed (Morrell et al., 2003; Kumar et al., 2008). These structural changes may explain persistent memory impairment among patients with OSA even after treatment, and possibly could partly account for cognitive issues among TLE patients with OSA.

There is also increasing evidence that treatment of OSA leads to better seizure control among patients with DRE (Rashed et al., 2019). However, this has not been specifically addressed for TLE.

There was some evidence to support the relationship between TLE and sleep apnea in our review, with varying prevalence. None of the studies described risk factors for higher prevalence of OSA in TLE specifically. The differences in OSA prevalence observed in these studies may be consequent to several factors, not yet systematically addressed, e.g., mean age, ethnicity, and epilepsy characteristics. Since risk factors for common sub-groups of TLE like MTLE-HS are largely acquired (antecedents like febrile status epilepticus in early life), rather than genetic, any direct pathophysiological linkage with OSA (e.g., craniofacial malformations) is unlikely. The association with epilepsy co-morbidities and medication effects like obesity is more likely (Foldvary-Schaefer and Grigg-Damberger, 2006; Sivathamboo et al., 2019).

#### Sleep and Cognition in Temporal Lobe Epilepsy

Sleep disturbances are common among persons with TLE, as are memory deficits, which are the most frequently encountered cognitive issue. The interaction between sleep and memory impairment in TLE has been assessed in very few clinical studies (Table 6). In a study by Miller et al. (2016) among 16 patients with TLE who underwent ambulatory EEG, patients spent shorter time in slow wave sleep and demonstrated prolonged REM latency. Memory complaints were assessed *via* the Everyday Memory Questionnaire (EMQ). However, no clear relationship could be observed between memory complaints and sleep parameters including time in slow wave sleep, time in REM sleep, and time awake after sleep onset. However, prolonged REM latency was associated with memory impairment. In another study by van Schalkwijk et al. (2018) on the same group of patients, the percentage of slow wave sleep was associated with next day verbal learning, longer REM latency was associated with poorer retention of autobiographical events (AE) memory, which was worse for patients with TLE compared to ETLE. Slow wave sleep correlated positively with word-list learning. The presence of hippocampal lesions was associated with poorer performance on memory scores. Regression analysis indicated that the presence of a hippocampal lesion was more important than REM latency in predicting overnight AE retention, inviting the question whether abnormal REM latency was merely a biomarker of hippocampal pathology. Chakravarty et al. (2019) compared the association of sleep and cognitive and memory function among persons with refractory and medically controlled focal epilepsy. Of the refractory group, 51.35% (n-19) patients had TLE. While sleep disturbances were observed with a higher frequency in the refractory group, which included shorter actual total sleep time, longer self-reported total sleep time, poorer sleep efficiency and prolonged sleep and REM latency, positive correlation was noted also between higher total sleep time and sleep efficiency with memory/executive function. However, this study has not provided analysis of TLE versus ETLE subgroups.

**TABLE 6:** Studies assessing impact of sleep on memory and cognition among patients with TLE.

Author/Year	Study design	Number of patients	Instruments used for cognitive evaluation	Instruments used to evaluate sleep	Outcomes
Miller et al., 2016	Cross-sectional	25 with focal epilepsy (16 TLE)	EMQ	Ambulatory EEG	Longer REM latency associated with poorer everyday memory
van Schalkwijk et al., 2018	Cross-sectional	25 with focal epilepsy (16 TLE)	AE memory, verbal memory, visuospatial memory, verbal learning	Ambulatory EEG	Longer REM latency and presence of hippocampal lesion associated with poorer retention of AE memory Negative correlation between % SWS and overnight retention of RCFT
Chakravarty et al., 2019	Prospective observational	37 with refractory focal epilepsy, 37 with medically controlled focal epilepsy, and 40 age and sex-matched controls	Western Aphasia Battery, PGI memory scale, Trail making tests A and B, Digit symbol test, Stroop task, Verbal fluency test	PSG, structured sleep questionnaire, ESS, PSQI	Shorter total sleep time, longer sleep latencies, poorer sleep efficiency associated with poorer cognitive function in patients with refractory epilepsy

*AE, autobiographical events; EEG, electroencephalography; EMQ, Everyday Memory Questionnaire; ESS, Epworth sleepiness scale; PSG, polysomnography; REM, rapid eye movement; RCFT, Rey complex figure test; SWS, slow wave sleep; TLE, temporal lobe epilepsy.*

Overall, literature supports the crucial role of sleep, particularly REM sleep, in the synaptic consolidation of memory (Peigneux et al., 2004; Rudoy et al., 2009; Diekelmann and Born, 2010). Sleep preferentially reactivates recent representations in the mesial temporal lobes, which are further strengthened during slow wave sleep, for declarative memory. Two studies noted significant correlation between REM latency and memory in patients with TLE; longer REM latency was associated with poorer everyday memory as sampled by a questionnaire, and poorer retention of AE memory (Miller et al., 2016; van Schalkwijk et al., 2018). The study by Chakravarty et al. (2019) noted that though there was a positive association between REM latency and memory, it did not achieve statistical significance. The relationship between REM latency and memory is unclear. Longer REM latency has also been observed in patients with Alzheimer disease; among patients with epilepsy, nocturnal seizures, IEDs (Bazil et al., 2000), and hippocampal abnormalities (Miller et al., 2016) showed an association. IEDs may affect spindle formation, and thereby, impact hippocampal-neocortical connections, relevant for memory consolidation (Frauscher et al., 2015). Whether REM latency is simply a marker of disordered sleep, or a causative mechanism for memory impairment in TLE certainly deserves further exploration. More interesting associations are currently being explored, hence, future research in this area promises to yield much deeper understanding of specific sleep cognition relationship in TLE and epilepsy in general. A recent study, published in abstract form demonstrated K complex associated sleep spindle density to be least among TLE patients with both memory and language impairment, with overall spindle density being significantly lower among those with only memory impairment compared to those with only language impairment (Gupta et al., 2019).

#### Sleep and Mortality in Temporal Lobe Epilepsy

There are potential links between sleep and sudden death among patients with TLE. Among toddlers, significantly higher proportion of children with sudden unexplained death were found dead after a sleep period, compared to children without sudden death, or other established causes of death (98% versus 43%; *p* < 0.001) (Do et al., 2019). Hippocampal abnormalities were more prevalent in the group of children with sudden death compared to control group (62% versus 22%; *p* = 0.06), although this was not statistically significant. Children with sudden death with hippocampal abnormalities were also more likely to be found in the prone position. These findings generated the hypothesis that sudden death may be consequent to an unwitnessed seizure emanating from the temporal lobe, possibly leading to downstream effects on cardiac/respiratory systems combined with airway occlusion due to face down position. In another study by Nayak et al. (2017) lack of heart rate variability in patients with drug-naïve TLE suggested abnormal reflex baroreceptor activation, which may predispose them to SUDEP. Lateralization in TLE also has implications for SUDEP (Dono et al., 2020). Left TLE, compared to right TLE, is reported to be associated with increased vagal tone, and may be therefore associated with lower risk of cardiac dysfunction and SUDEP.

### Treatment Implications

#### Effects of Antiseizure Medications

Antiseizure medications may have significant effects on sleep architecture in patients with epilepsy (Jain and Glauser, 2014). Valproic acid, high dose levetiracetam, and phenobarbital increase daytime somnolence. Pregabalin, gabapentin, tiagabine, clobazam and carbamazepine reduce sleep latency and may increase sleep efficiency. Although most of these studies have involved patients with focal epilepsy, there are very few focusing on TLE alone. The study by Nayak et al. (2016) among patients with TLE showed that carbamazepine worsened sleep parameters, *via* increased CAP alterations, thereby increasing sleep instability. In another study by Romigi et al. (2020), eslicarbazepine was found to improve sleep continuity among patients with TLE.

#### Epilepsy Surgery

Epilepsy surgery may lead to improvement in sleep parameters (Table 7). This could be attributable to reduction in seizure frequency. This was clear from the study by Zanzmera et al. (2013) which showed improvement in sleep quality only among those with successful epilepsy surgery.

**TABLE 7:** Studies assessing the impact of epilepsy surgery on sleep parameters among patients with TLE.

Author/year of publication	Type of study	Number of patients who underwent surgery	Study instruments used	Outcomes
Carrion et al., 2010	Prospective observational	59	ESS, PSQI	ESS and PSQI scores reduced significantly after surgery
Serafini et al., 2012	Prospective observational	11	VEEG	Follow up: 1 and 2 years after surgery TST and REM increased after surgery
Zanzmera et al., 2013	Prospective observational	17	ESS, PSG	At 3 months, patients with good surgical outcome showed significant improvement in self-reported sleep parameters and seizure frequency but not among patients with poor surgical outcome
Yaranagula et al., 2021	Prospective observational	20	ESS, PSQI, NCSDQ, PSG	Assessed at 3 months- no significant effect of epilepsy surgery on sleep parameters; mild reduction in ESS and PSQI scores.

*ESS, Epworth sleepiness scale; NCSDQ, NIMHANS Comprehensive Sleep Disorders Questionnaire; PSG, polysomnography; PSQI, Pittsburg Sleep Quality Index; REM, rapid eye movement; TST, total sleep time; VEEG, video electroencephalography.*

In a controlled study by Carrion et al. (2010), 59 patients with TLE-DRE were evaluated for change in ESS and PSQI 3 months and 1-year post-epilepsy surgery. ESS scores and mean global PSQI scores were found to decrease significantly after surgery, although ESS scores were not in the abnormal range in any case. Both ESS and PSQI assess subjective sleep experience, and objective parameters were not evaluated in this study. In another study by Serafini et al. (2012) total sleep time and REM sleep improved following epilepsy surgery at 1 and 2 years. However, Yaranagula et al. (2021) did not observe any significant change in sleep parameters after epilepsy, although follow up in this study was short, with mean follow up of around 5 months.

#### Vagus Nerve Stimulation

Although a few studies have assessed effects of vagus nerve stimulation (VNS) among patients with refractory epilepsy (Malow et al., 2001; Galli et al., 2003; Hallböök et al., 2005; Ravan and Begnaud, 2019), including small numbers of TLE patients, no studies have specifically assessed effects of VNS in TLE.

Vagus nerve stimulation is also known to trigger sleep disordered breathing, dependent on stimulation settings (Romero-Osorio et al., 2018; Salvadé et al., 2018; Vespa et al., 2021).

#### Ketogenic Dietary Therapy

Ketogenic diet has been shown to improve sleep quality and sleep anxiety among children with epilepsy (Hallböök et al., 2007; Ünalp et al., 2021). Studies among TLE patients are lacking.

#### Targeting Sleep for Improving Epilepsy Outcomes

There are preliminary reports of improved sleep quality and efficiency through treatment of sleep disorders like OSA resulting in better seizure control among patients with epilepsy in general, but this has not been studied specifically in TLE. A meta-analysis reported higher prevalence of OSA among PWE, compared to healthy controls, although there was no significant difference among DRE compared to controlled epilepsy (Lin et al., 2017). Continuous positive pressure airway (CPAP) treatment led to improved seizure control, compared to untreated patients (Odds ratio 5.26; 95% CI 2.04–13.5).

We found very few studies specifically investigating effects of different ASMs on sleep in TLE, hence specific mechanisms of effects linked with TLE cannot be commented upon.

Seizure freedom through epilepsy surgery (and potentially other interventions) may lead to improvement in subjective and objective sleep parameters. However, this may partly be due to reduction in seizure frequency following surgery. The exact role of epilepsy surgery apart from seizure reducing properties, deserves further consideration.

We found no studies to assess effects of ketogenic diet or vagus nerve stimulation on sleep parameters in TLE. Similarly, much more work is required to study the effect of targeting sleep to improve epilepsy outcomes systematically in TLE.

### Strengths and Limitations

We included studies from the year 2000 onward, and hence, compiled most recent literature, given the rapid advancement of knowledge accumulated over the last two decades. This is the main strength of this review. A major corpus of literature on sleep and epilepsy relationship stems from data prior to 1999, and although several reviews have attempted to study this relationship, none have focused on TLE *per se*.

Our review was limited to studies which included well-defined populations with TLE, and we mostly excluded studies on epilepsy in general, although TLE was likely to have formed a large proportion of these patients. There is a possibility of missing some important pointers toward mechanisms highlighting the sleep-TLE-relationship, due to the heterogeneity and non-specific inclusion criteria of many of these studies. Nevertheless, our stringent inclusion of studies focused on TLE has strengthened the review.

### Future Directions for Research

Effects of different ASMs on sleep in patients with TLE could inform treating physicians much better regarding medication choice. This needs to be studied in more prospective studies on commonly used ASMs working through different mechanisms of action.

Another major aspect that requires detailed investigation in this field, is specific sleep related targets for improvement in outcomes among patients with TLE. Our group is currently working on completion of analysis of a recently concluded randomized trial (CTRI/2017/04/008359), which could potentially fill this gap to quite an extent.

Additionally, the complex relationship of mortality risk, specifically SUDEP with DRE-TLE requires urgent exploration as well. More work is needed in identification of sleep related targets for reducing secondary generalization in seizures among patients with TLE, as this could directly help reduce SUDEP risk.

## Conclusion

Temporal lobe epilepsy and sleep share a fascinating and often, reciprocal relationship. Sleep may be utilized to enhance the diagnostic yield of seizures and IEDs, both *via* NREM cycles, as well as sleep deprivation. Sleep is also a major trigger for secondary generalization of seizures in TLE, which has clinical implications. Reciprocally, TLE is associated with self-reported excessive daytime somnolence, with reduced actual total sleep time, and poorer sleep related quality of life. Disruptions in sleep micro- and macroarchitecture have now been studied well in patients with TLE and clearly appear to contribute. Initial work exploring the sleep-cognition-epilepsy interface in TLE has already demonstrated important associations. More future work investigating the complex aspects of the role of sleep in seizure and non-seizure outcomes, as well as mortality reduction in TLE promises to unravel many yet undiscovered linking mechanisms.

## Author Contributions

DG performed the literature search, extracted and analyzed the data, and drafted the manuscript. LC performed the literature search, extracted and analyzed the data, and provided critical revisions. GS designed the review, performed the literature review, extracted and analyzed the data, provided the critical revisions, and steered the manuscript. All authors contributed to the article and approved the submitted version.

## Conflict of Interest

The authors declare that the research was conducted in the absence of any commercial or financial relationships that could be construed as a potential conflict of interest.

## Publisher’s Note

All claims expressed in this article are solely those of the authors and do not necessarily represent those of their affiliated organizations, or those of the publisher, the editors and the reviewers. Any product that may be evaluated in this article, or claim that may be made by its manufacturer, is not guaranteed or endorsed by the publisher.

## Abbreviations

AHI: apnea hypopnea index
ASM: antiseizure medication
CAP: cyclic alternating pattern
CBZ: carbamazepine
DRE: drug refractory epilepsy
EEG: electroencephalography
ETLE: extratemporal lobe epilepsy
FLE: frontal lobe epilepsy
FoE: foramen ovale electrode
HS: hippocampal sclerosis
IED: interictal epileptiform discharges
MTLE: mesial temporal lobe epilepsy
NCSDQ: NIMHANS Comprehensive Sleep Disorders Questionnaire
NREM: non-rapid eye movement
OSA: obstructive sleep apnea
PLM: periodic limb movements
PPTg: pedunculopontine tegmental nucleus
PSG: polysomnography
PSQI: Pittsburgh Sleep Quality Index
REM: rapid eye movement
SSS: Stanford sleepiness scale
STN: subthalamic nucleus
SUDEP: sudden unexplained death in epilepsy
TIB: time in bed
TLE: temporal lobe epilepsy
TST: total sleep time
WASO: wake after sleep onset.
